# Hydrophilic Excipient-Independent Drug Release from SLA-Printed Pellets

**DOI:** 10.3390/pharmaceutics13101717

**Published:** 2021-10-17

**Authors:** Lei Xu, Qingliang Yang, Wei Qiang, Huijie Li, Weizhen Zhong, Siying Pan, Gensheng Yang

**Affiliations:** 1 College of Pharmaceutical Science, Zhejiang University of Technology, Hangzhou 310014, China; leixuzjut@163.com (L.X.); weiq1kcl@gmail.com (W.Q.); lihjie2019@163.com (H.L.); zwzzhong@163.com (W.Z.); psying2020@163.com (S.P.); 2 Research Institute of Pharmaceutical Particle Technology, Zhejiang University of Technology, Hangzhou 310014, China

**Keywords:** stereolithography, three-dimensional printing, 3D printed oral dosage forms, mini-sized pellets, modified release

## Abstract

Three-dimensional (3D) printing technology, specifically stereolithography (SLA) technology, has recently created exciting possibilities for the design and fabrication of sophisticated dosages for oral administration, paving a practical way to precisely manufacture customized pharmaceutical dosages with both personalized properties and sustained drug release behavior. However, the sustained drug release achieved in prior studies largely relies on the presence of hydrophilic excipients in the printing formulation, which unfortunately impedes the printability and formability of the corresponding printing formulations. The current study developed and prepared mini-sized oral pellets using the SLA technique and successfully accomplished a hydrophilic excipient-independent drug release behavior. With ibuprofen as the model drug, the customized photopolymerizable printing formulation included polyethylene glycol diacrylate (PEGDA) as a monomer and diphenyl (2,4,6-trimethylbenzoyl) phosphine oxide (TPO) as a photoinitiator. The produced mini-sized pellets were thoroughly investigated for various factors, including their printability, physical properties, microscopic features, drug content, and drug-release profiles. The drug release profiles from the printed pellets that were larger size (3 mm and 6 mm) followed the Ritger–Peppas model, demonstrating that the release was influenced by both the diffusion of the dissolved drug and by the erosion of the hydrophilic excipients (PEG400). The profiles from the smaller printed pellets (1 mm and 2 mm) followed first release kinetics, not only illustrating that the release was impacted only by drug diffusion, but also indicating that there is a size boundary between the dependent and independent hydrophilic excipients. These results could create practical benefits to the pharmaceutical industry in terms of the design and development personalized dosages using the SLA printing technique with controllable drug release by manipulating size alone.

## 1. Introduction

Three-dimensional (3D) printing is a transformative additive manufacturing technology that allows the fabrication of objects from a computer-aided design (CAD) file in a layer-by-layer manner [1]. Owing to its brilliant capability to generate a complex structure with precise geometries, it has recently attracted extraordinary attention in not only medical fields [2,3] such as tissue engineering [4,5], dental prothesis [6,7], and biomedical implants [8,9,10], but also in pharmaceutical areas. Specifically, 3D printing could potentially create extra benefits in terms of both personalized design and precise fabrication of drug dosages with sophisticated geometries and programmable drug release behaviors, including immediate release [11,12] or modified drug release profiles [13,14,15], some of which may contain multiple drugs [16,17]. The most widely applied 3D printing techniques include fused deposition modeling (FDM) [18,19,20,21,22] and printing technologies based on photopolymerization. One of the most attractive advantages of the FDM process is its exceptional adaptiveness to all pharmaceutical actives and excipients as long as they are adequately thermally stable. However, the relatively low precision and the discontinuous printing process (the requisite of an additional step for the preparation of the printing filament) of the FDM currently impede its practical applications, specifically those for temperature-sensitive drugs and ingredients [23,24].

Recently, photopolymerization printing technology has increasingly attracted a great amount of attention owing to its superior printing accuracy, improved feature resolution, and the high quality of the final products. According to the classification of 3D printing technology by The American Society for Testing and Materials (ASTM), photopolymerization printing techniques are mainly divided into two categories: material jetting and vat photopolymerization [25]. Commonly used vat photopolymerization techniques include stereolithography (SLA), digital light processing (DLP), continuous liquid interface production (CLIP), and two-photon polymerization (2PP), all of which involve a process utilizing light irradiation (e.g., laser beam, UV, and visible light) to create solid objects from a photoreactive liquid resin [26,27]. Thanks to their appealingly high precision and continuous processing at a small scale, these techniques have been proposed for the development and fabrication of more sophisticated and complicated dosages, such as transdermal microneedles [28] and bio-inspired drug delivery systems [29]. The SLA technique is the most widely applied photopolymerization printing technique due to its high resolution and low overall cost; recently, it has been successfully utilized to produce solid oral dosage forms such as tablets [30,31,32], hydrogels [33,34,35], and polypills [36,37].

However, one of the crucial limitations of SLA-printed dosages is the potential incomplete drug release caused by the entrapment of active ingredients within the polymeric matrix after polymerization and the resulting low molecular mobility. Basit et al. revealed that the drug content of printed printlets was obviously decreased and that the absolute amount of drug decreases as the monomer content increases [13]. Additionally, it has been previously proven that printed solid dosages with a higher content of stereolithography monomer show a lower dissolution rate because of the higher degree of cross-linking that occurs in the produced dosage matrix. Gaisford et al. found that the dissolution rate of different drugs in different pellet forms slowed down with the increase of PEGDA [33]. To address this challenge, normally hydrophilic excipients were necessarily added into the liquid resin as porogens (pore formers) prior to the printing process, producing channels within the polymer matrix for more acceptable drug release. Unfortunately, the presence of those hydrophilic excipients in the photopolymer resin could possibly result in the unacceptable printability and low mechanical properties of the printed dosages due to the non-photocurable nature of such excipients [38]. This is particularly true when the ratio of the applied porogens is high or even at a surplus. Consequently, a careful balance needs to be reached between the concentration of the hydrophilic excipients for a sufficient pore ratio that maintains the acceptable printability and mechanical properties of the printed dosages. The present study aims to investigate the possibility of 3D printing oral solid dosages with a desirable drug release behavior without use of hydrophilic excipients by using the SLA technique and to explore the influence of dosage size on the drug release rate.

## 2. Materials and Methods

### 2.1. Materials

Poly(ethylene glycol) diacrylate (PEGDA600, average MW 700), polyethylene glycol (PEG, average MW 400), diphenyl (2,4,6-trimethylbenzoyl) phosphine oxide (TPO), ibuprofen, and tartrazine were purchased from Aladdin Chemicals Ltd. (Shanghai, China). All salts required in the preparation of the dissolution media were also produced from Aladdin Chemicals Ltd. (Shanghai, China). All other ingredients and chemicals used were of analytical grade or higher.

### 2.2. Preparation of Photopolymer Solution

PEGDA600 and PEG400 were used to prepare the photoreactive solutions; TPO was used as the photoinitiator (PI). PEGDA600 and PEG400 were mixed according to the predesigned ratios (Table 1) for 1 h, and then TPO was added into the mixture, and the whole solution was stirred for at least 8 h at 25 °C until the TPO was completely dissolved. Then, the model drug (ibuprofen) was added to the solution at a concentration of 10% (*w*/*w*) and was mixed thoroughly for the complete dissolution of the drug. Additionally, in order to investigate the printability of the pellets, a typical photoabsorber (PA), tartrazine, was also added into the solution, and the mass ratio of PEGDA600 ranged from 0% to 0.12% (*w*/*w*, ratio to mass of PEGDA600 based on the Formulation 1 in Table 1). The whole process was protected from light. Then, the prepared photopolymer solution was loaded into the resin tank of the printer.

### 2.3. Printing Pellets

All of the pellets were fabricated with a commercial Formlabs Form 2 SLA 3D printer (Formlabs Inc., Somerville, MA, USA) equipped with a 405 nm wavelength light source. The SLA printer is capable of fabricating small objects with a high resolution of 140 μm and layer thicknesses of 25, 50, 100 μm. The pellet models, which were spheres with diameters of 1 mm and 2 mm, were designed using the engineering software SolidWorks 2018 (Dassault Systems, Waltham, MA, USA) and were exported as a stereolithography (.stl) file into the 3D printer software (Preform Software v.3.1.2, Formlabs, UK). “Open Mode” was enabled on the printer to allow the printing of third-party resins.

### 2.4. Printability Evaluation 

The influence of photoabsorber of tartrazine on the printability of the pellets was investigated by using the divergences, specifically the roundness, between the average measured properties and the theoretical properties that were reported by Fatouros and co-workers [14]. Roundness was defined as the difference between the long diameter and short diameter of the printed pellets; the long diameters and short diameters of all of the pellets were measured using calipers (*n* = 10).

### 2.5. Characterization of Printed Pellets

#### 2.5.1. Physical Properties/Evaluation

The diameters (including the long diameter and the short diameter) of the printed pellets were measured using slide calipers (*n* = 10). The mean diameter was defined as the average of the shortest and longest distances of each pellet. Additionally, the weight of each pellet (*n* = 10) was evaluated using a Sartorius analytical balance with a resolution of 1 × 10^−5^ g. Row means with standard deviation (S.D.) were used to analyze the variance between the pellet diameters, and a t test was used to determine the significant difference between the model and printed pellets, with differences being considered significant when *p* < 0.05.

#### 2.5.2. Tensile Strength 

The breaking force of the printed pellets was measured using a texture analyzer (TA. XT Plus, SMS, UK) with diameters of 1 mm and 2 mm for each formulation (*n* = 10); the tensile strength of the pellets was calculated according to the following Equation, Equation (1) [35]:

(1) $$\delta =\frac{2F}{\pi D^{2}}$$

where *δ* is the tensile strength, *F* is the pellet breaking force (load), and *D* is the diameter of the pellets.

#### 2.5.3. X-ray Powder Diffraction (XRD)

The SLA printed pellets together with the sample of pure ibuprofen, and the results of the stability tests performed on the pellets were recorded using an X-ray powder diffraction. The X-ray powder diffraction was determined in a X’Pert PRO (PNAlytical, Almelo, Netherlands) using a Cu Ka X-ray source (λ = 0.1541 nm). The intensity and voltage applied were 15 mA and 40 kV. Samples were scanned from Theta = 3° to 40° at a step of 0.02° and a scan speed of 0.35 s/step.

#### 2.5.4. Scanning Electron Microscopy (SEM)

The surface and cross section images of the printed pellets before and after dissolution tests were taken with a scanning electron microscope (SEM, Hitachi S-4700 Hitachi High-Tech Science Corporation, Tokyo, Japan). Samples were fixed onto stubs with double sided adhesive tape and were sputter coated with gold under vacuum in an argon atmosphere prior to observation. The voltage was set at 15 kV under high vacuum condition.

#### 2.5.5. Drug Content 

To determine the drug content in both the photopolymer resin and in the 3D printed pellets, 100 mg of a drug-loaded formulation solution was diluted with 500 mL phosphate buffer (pH = 7.2) and was mixed thoroughly, while the printed pellets were crushed using a mortar and pestle, with the crushed pellets being transferred into a 500 mL volumetric flask and then diluted with phosphate buffer (pH = 7.2). The solution was sonicated for 30 min and kept with constant magnetic stirring overnight at room temperature. Samples of the solutions were then filtered through 0.45 μm filters (Millipore Ltd., Ireland), and the drug concentration was determined with a spectrophotometer (UV-2450; Shimadzu, Tokyo, Japan) at 222 nm based on the valid ibuprofen calibration curve (A = 0.04669*C* − 0.00413, R = 0.9991).

### 2.6. Dissolution Tests of Printed Pellets

Drug dissolution tests of the printed pellets were conducted with a USP-II apparatus (Apparatus 2, paddle; Tianda Tianfa Technology Co., Ltd., Tianjin, China). Phosphate buffer solution with a pH value of 7.2 was chosen as the release media. The paddle speed of the USP-II was fixed at 50 rpm, and the tests were conducted at 37 ± 0.5 °C (*n* = 6). At the predetermined time intervals, the 10 mL dissolution medium was withdrawn and was replaced with fresh medium. The withdrawn sample was filtered through a 0.45 μm membrane filter. The percentage of drug released from the pellets was determined using the HPLC (Ultimate 3000, ThermoFisher, Walthman, MA, USA) method; the validated HPLC assay entailed injecting 20 μL samples for analysis, using a mobile phase with an isocratic elution system of (A) acetonitrile and (B) 0.1% sodium acetate in distilled water, through an DIONEX 5 μm-C18 150 × 4.6 mm (ThermoFisher, Walthman, MA, USA), maintained at 35 °C. The mobile phase was pumped at a flow rate of 1 mL/min under the following gradient program: 60% A; the eluent was screened at a wavelength of 263 nm, and the retention time was 6.2 min.

#### Drug Release Kinetic Profile 

The release kinetics of the ibuprofen from the SLA-printed pellets was investigated by fitting the cumulative drug release data to various mathematical models including zero-order, first-order, Higuchi, and Ritger–Peppas models [39] (Equations (2)–(5)). The correlation coefficients (R) of the fitted equations were recorded using the DDSolver software (Microsoft Excel add-in program) developed by Zhang et al. [40].

(2) $$\mathrm{Zero}\;\mathrm{order}:\;\frac{Mt}{M\infty }=K_{0}\cdot t$$

(3) $$\mathrm{First}\;\mathrm{order}:\;\frac{Mt}{M\infty }=100\left[1-\mathrm{Exp}\left(-K_{1}\cdot t\right)\right]$$

(4) $$\mathrm{Higuchi}\;\mathrm{model}:\;\frac{Mt}{M\infty }=K_{H}\cdot t^{1/2}$$

(5) $$\mathrm{Ritger}-\mathrm{Peppas}:\;\frac{Mt}{M\infty }=K_{p}\cdot t^{n}$$

(6) $$\mathrm{Hixson}-\mathrm{Crowell}:\;{\left(100-\frac{M_{t}}{M_{\infty }}\right)}^{1/3}=-K_{c}\cdot t$$

where *Mt/M∞* is the fraction (%) of drug released at time *t*; *K*_0_, *K*_1_, *K_H_, K_P,_ K_c_* are drug release constants for the corresponding models; and *n* is the release exponent, indicating the drug release mechanism.

### 2.7. Stability Study of Printed Pellets

Printed pellets (1 mm and 2 mm) with different formulations were placed in HDPE vials (50 mL) and sealed with aluminum film and were then stored at 40 °C /75% RH for 1 month. The dissolution tests of these pellets before and after storage were examined.

### 2.8. Statistical Analysis

The experimental results were expressed as mean ± Standard Deviation (S.D.) values. Additionally, the similarity factor (*f*_2_) was applied to compare the drug release profiles before and after storage in the stability tests, which can be calculated using Equation (7).

(7) $$f_{2}=50\cdot \log\left\{{\left[1+\frac{1}{n}\overset{t}{\underset{n=1}{\sum }}{\left(R_{t}-T_{t}\right)}^{2}\right]}^{2}\cdot 100\right\}$$

In this Equation, *n* is the total number of sampling times, and *R_t_* and *T_t_* are the accumulated drug release percentages at time point t for the reference and test products, respectively. The value of *f*_2_ is between 0 and 100, and if *f*_2_ is larger than 50 (between 50 and 100), then these two release profiles are considered to be similar. On the other hand, if *f*_2_ is close to 100, the two release profiles are considered to be identical.

## 3. Results and Discussion

### 3.1. Printability Evaluation 

**Figure 1:**
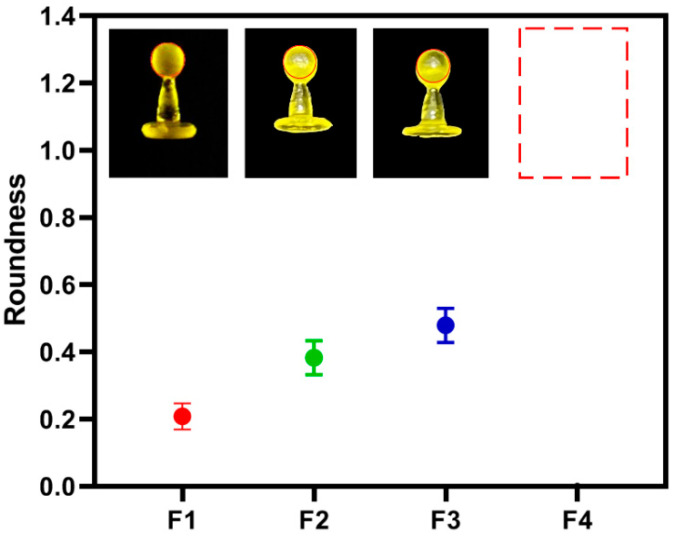
The roundness of printed pellets with different formulations and 0.10% tartrazine concentration.

Adequate printability is a prerequisite for the applied printing materials to create a mechanically acceptable product. Essentially, in the SLA process, the printing materials necessarily contain photocurable monomers and photoinitiator (PI) for photopolymerization and stereolithography. Additionally, photoabsorber (PA) is also commonly applied in the printing formulation to avoid undesirable curing and to eliminate the over-curing of the non-printed area during the printing process, considerably improving the printability of the corresponding printlets [41]. The present study adopted tartrazine as the PA due to its excellent solubility and non-toxicity (safe dosage: 7.5 mg·kg^−1^ bw/day) [42] and investigated its effect on the printability of the pellets by evaluating their roundness. As shown in Table 2, with the increase of the tartrazine ratio (*w*/*w*) from 0% to 0.1% in the printing formulation, the roundness of the printed pellets decreased from 1.90 to 0.18, indicating an increasingly reduced undesirable curing area and enhanced printability. While further increasing the tartrazine ratio to 0.12% resulted the incomplete curing of photopolymer, leading to the printed pellets having a shorter diameter than the model size. Consequently, the optimized tartrazine ratio is 0.10%, based on the mass of PEGDA600, and it was applied to the following experiments. Additionally, the influence of the hydrophilic excipient (PEG400) on the printability of the pellets was also characterized. As shown in Figure 1, an increase of the PEG400 from 0% to 50% in the printing formulation led to an increase of the roundness from 0.18 to 0.48, demonstrating a reduction in the printability of the corresponding pellets. Additionally, further increasing the PEG400 ration to 70% (formulation 4) caused a printing failure. These results illustrate that the presence of PEG400 strongly reduces printability during the SLA printing process.

### 3.2. Physical Propertiesof the Printed Pellets

**Figure 2:**
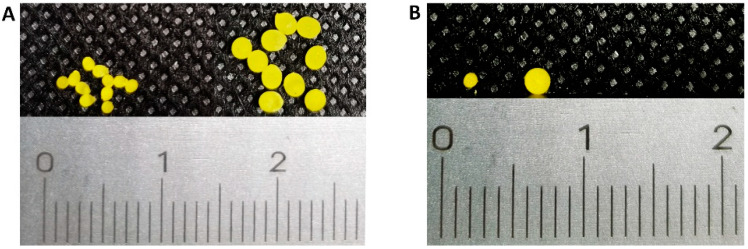
(**A**) Front-view of 1mm and 2mm pellets. (**B**) Top-view of each sized pellets.

As shown in Figure 2, ibuprofen-loaded pellets were successfully fabricated by the SLA 3D printing technique. The yellow color of these pellets came from the photoabsorber of tartrazine. Ten pellets were randomly selected from different batches with different formulations, and their appearance showed good uniformity and consistency.

**Figure 3:**
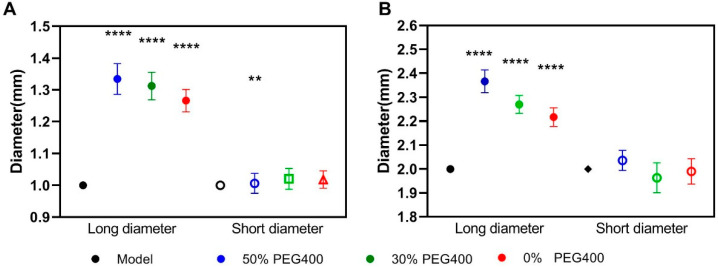
Diameter uniformity of pellets fabricated with different formulations (*n* = 10). (**A**) The long and short diameters of 1 mm pellets with different formulations; (**B**) the long and short diameters of 2 mm pellets with different formulations. (** = *p* < 0.01, **** = *p* < 0.0001).

Figure 3 shows the diameter of the printed pellets, illustrating the adequate accuracy and repeatability of the SLA printing process. Additionally, the presence of the PEG400 in the printing formulation had a big impact on the size variability of the printed pellets, both for the 1 mm (Figure 3A) and 2 mm (Figure 3B) pellets. Compared to the designed dimensions, there were no significant differences (*p* > 0.05) between the short diameter in the formulation with 0% PEG400 and in the formulation with 30% PEG400. For the formulation containing 50% PEG400, the short diameter showed a slight difference (Figure 3A) in the 1 mm pellets, which indicated that the printing accuracy might be influenced by hydrophilic excipients, especially when it was at a high ratio in the photopolymer printing solution. However, it was found that each group displayed significant differences (*p* < 0.0001) in terms of their long diameters when compared to the model, which might be caused by either the presence of the hydrophilic PEG400 or by the incomplete peeling of the support structure during the post-printing process. Additionally, we also found that when increasing the content of PEG400 to 70%, the printing failure would occur in both the 1 mm and 2 mm pellets due to the worse physical properties of the materials caused by the high ratio of hydrophilic excipients in the formulation.

**Figure 4:**
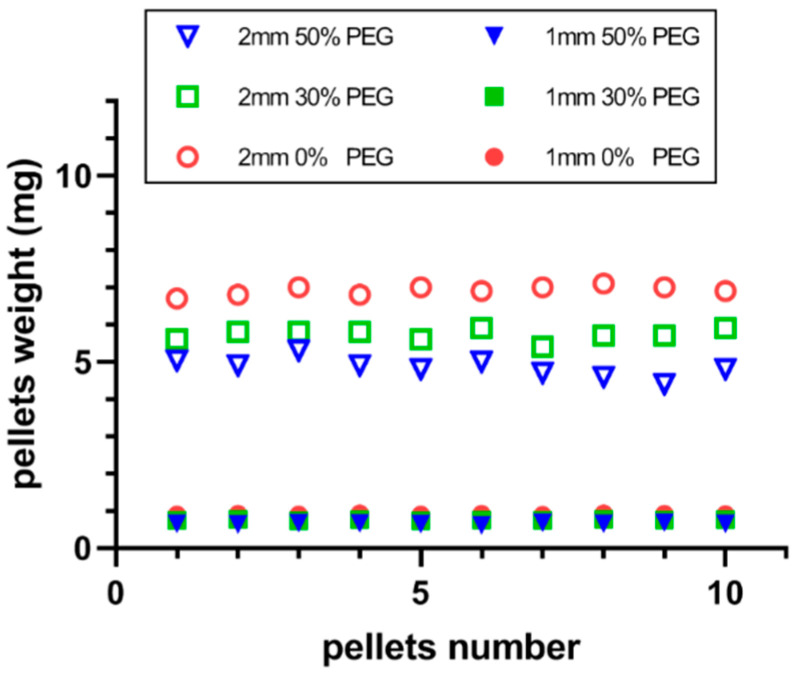
Weight uniformity of the printed pellets with different formulations.

Additionally, the weight uniformity of the printed pellets is demonstrated in Figure 4 and was adequately consistent; there was no statistically significant differences (*p* > 0.05). The weight variation for each group of printed pellets was less than 5%, which fulfills the requirements of the USP specifications [43] with better precision than conventional manufacturing processes such as granulation or spheronization. However, the presence of the hydrophilic excipient (PEG400) also strongly impacted the absolute weight of the printed pellets, as increasing the content of PEG400 led to a decrease in the absolute weight of the pellets. This could be caused by the low density of the applied PEG400.

**Figure 5:**
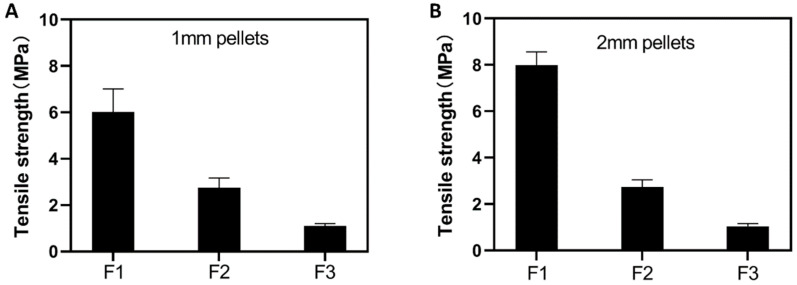
Tensile strength comparison of formulations with different content of PEG400. (**A**) 1 mm pellets; (**B**) 2 mm pellets (F1 contains 0% PEG400, F2 contains 30% PEG400, F3 contains 50% PEG400).

More importantly, the presence of the hydrophilic excipient PEG400 could also significantly impact the mechanical properties, particularly the tensile strength, of the printed pellets. As shown in Figure 5, the tensile strength of the printed pellets, both 1 mm (Figure 5A) and 2 mm (Figure 5B), dramatically decreased with the increase of the PEG400 content. It was reported that an acceptable tensile strength for the printed solid dosages should not be smaller than 1.7 Mpa [44], yet the tensile strength of the printed pellets containing 50% PEG400 was smaller than this value, which is considered to be unacceptable.

These results demonstrate that during the SLA printing process, the hydrophilic excipients applied in the printing formulation, such as PEG400, can strongly influence the physical and mechanical properties of the final products, hence their need to be strictly and carefully balanced.

### 3.3. Physical Form of the Applied Drug

**Figure 6:**
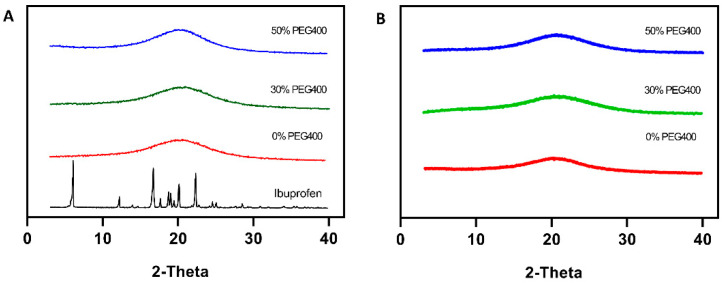
(**A**) X-ray powder diffractograms of the pure drug and printed pellets. (**B**) X-ray powder diffractograms of the printed pellets after the stability tests.

Physical forms of the applied drug (ibuprofen), both of the pure drug and of those in the polymer matrices after printing, were analyzed using XRD. As shown in Figure 6, pure ibuprofen exhibited multiple diffraction peaks at around 5°, 12°, and 20° 2θ, clearly reflecting that the active was in the crystalline form. However, there no peaks appeared in the patterns of the printed pellets for all the formulations, indicating that the SLA process adopted in the present study successfully altered the applied actives (ibuprofen) from crystalline to amorphous form, which is more desirable due to the significant improvement of the solubility. This could be potentially applied to enhance the solubility of the APIs from the Biopharmaceutical Class Systems (BCS) II category. After the stability tests, no peaks appeared in the pellets, illustrating that the amorphous drug is stable in the pellets and that there has not been any crystal transformation.

### 3.4. Morphology of the Printed Pellets

**Figure 7:**
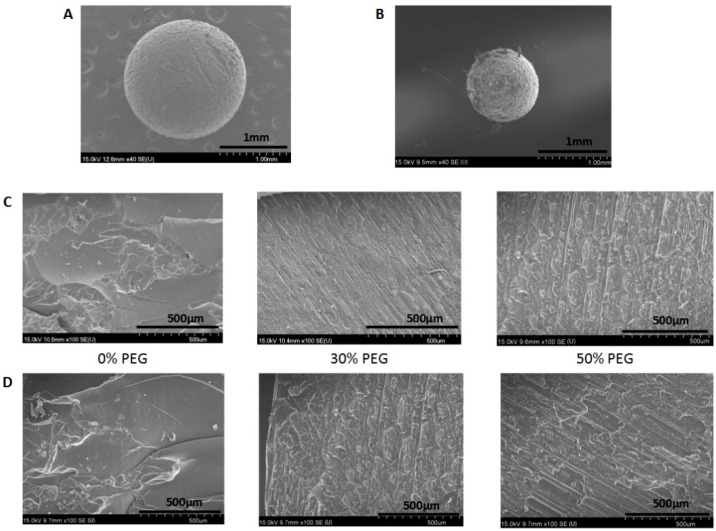
Scanning electron microscopy images of printed pellets: (**A**) overall morphology of the 2 mm pellets; (**B**) overall morphology of the 1 mm pellets; (**C**) the cross-section of 2 mm pellets with different contents of PEG400; (**D**) the cross-section of 1 mm pellets with different contents of PEG400.

The morphologies of the SLA-printed pellets are shown in Figure 7 and include both the surface morphology (Figure 7A,B) and the cross-section images of the pellets with different printing formulations. Figure 7A,B illustrate that the pellets with a predesigned sphere shape were successfully printed with a rough surface, which resulted either from the layer-by-layer printing process or from the polymerization reaction. Additionally, the cross-section image of the pellets (Figure 7C,D) demonstrated that the internal structure of the printed pellets without PEG400 was homogeneous. Additionally, visible layers within the printed pellets formed during the printing process could be clearly observed in the cross-section images, and those layers became clearer when the content of the hydrophilic excipient (PEG400) in the formulation increased, with the surface became rougher and looser, which is consistent with the results of the tensile strength tests.

### 3.5. Drug Content and Uniformity

As shown in Table 3, the drug content in the photopolymer resin was sufficiently close to the theoretical value, and the slight reduction of the drug content in the pellets might be caused by the incomplete drug extraction from the drug–polymer crosslinked matrix, especially in the 2 mm pellets, in which the active drugs could be entrapped by the pellet matrix. This result is in accordance with previous studies [13,45]. There were no degradation peaks in the HPLC spectrum for the solution of the dissolved pellets, confirming that there was no significant drug loss during the printing process. This could be a potential benefit of the SLA printing technique over the fused deposition modeling (FDM) printing; the latter involves appreciable rises in temperature and has been shown to cause significant drug degradation when used to fabricate polymeric tablets [46].

### 3.6. Dissolution Analysis

**Figure 8:**
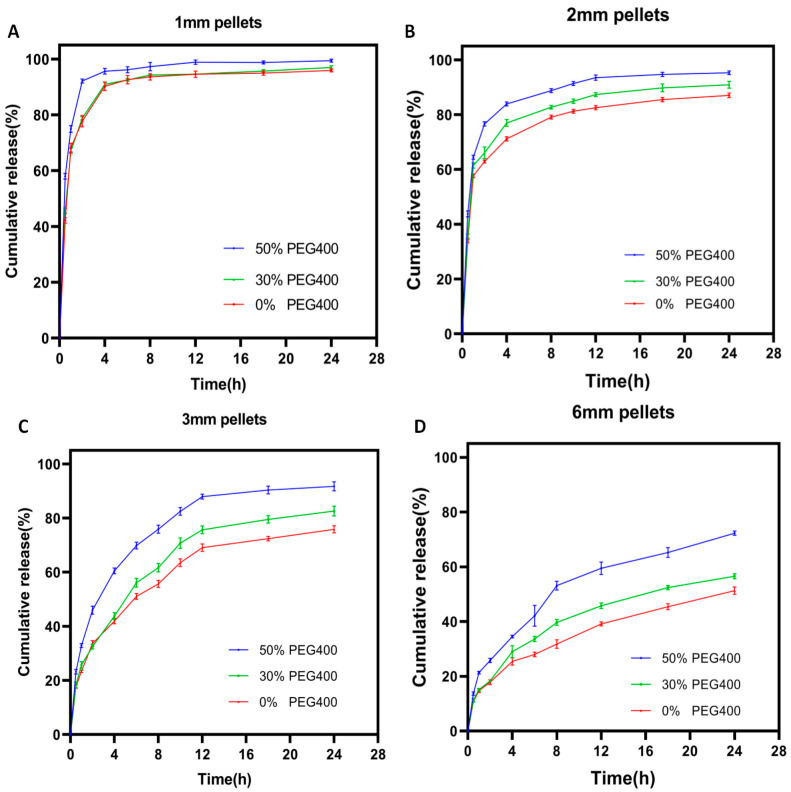
Drug dissolution profiles for different sized SLA-printed pellets: (**A**) 1 mm pellets; (**B**) 2 mm pellets; (**C**) 3 mm pellets; (**D**) 6 mm pellets.

Earlier attempts using the SLA technique to produce pharmaceutical dosages found that the drug release rate could be modified by either varying the concentration of hydrophilic excipients such as PEG [13] or by specifically designing the geometries of the prepared dosages [47]. However, the presence of these hydrophilic excipients in the printing formulation not only strongly influences the physical and mechanical properties of the printed dosages, but also potentially retard their printability and long-term stability. The present study found that the drug release rate from SLA-printed dosages could only be programmed by properly controlling the size of the printed pellets, which is favorable because of the hydrophilic excipient-independent drug release behavior. As shown in Figure 8, when the pellets were big (6 mm, Figure 8D), only 70% of the total drug was released from the printed pellets with the formulation containing 50% PEG400 after 24 h, which was further reduced with the decrease of the PEG400 ratio. Reducing the diameter of the printed pellets to 3 mm (Figure 8C) led to a faster drug release rate, but the hydrophilic excipient (PEG400) still played an important role in promoting the drug release rate. When further reducing the diameter of the pellets to 2 mm (Figure 8B) and 1 mm (Figure 8A), the drug released more quickly and more completely within 24 h. Additionally, the influence of the PEG400 on the release rate declined and was even eliminated with the 1 mm pellets.

**Figure 9:**
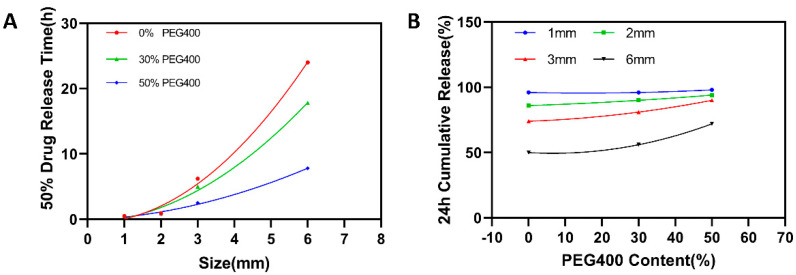
(**A**) Relationship between PEG400 content ratio (*w*/*w*), pellet size (mm), and 50% drug release time. (**B**) Relationship between PEG400 content ratio (*w*/*w*), pellet size (mm), and 24 h cumulative release content.

Figure 9A shows the relationship between the time needed for 50% drug release from the printed pellets and their sizes, clearly illustrating that the size of the printed pellets played a critical role in controlling the drug release rate, regardless of the printing formulation. While Figure 9B depicts the relationship between the drug cumulative release for 24 h and the concentration of the PEG400 in the printing formulation, the situations are obviously different. When the size of the pellets is bigger (6 mm and 3 mm), the increase of the PEG400 in the printing formulation significantly promotes the drug release rate. However, such influence disappeared when the reducing the diameter of the pellets to 2 mm and 1 mm. It can be concluded from those figures that the drug release rate from the SLA-printed pellets is not only desirably predictable, but is also precisely programmable by changing their size rather than by altering the composition of the printing formulation.

### 3.7. Drug Release Kinetics

The fitting results of the drug release profiles from SLA-printed pellets were shown in Table 4. For the larger pellets (3 mm and 6 mm), the release of ibuprofen followed the Ritger–Peppas model, indicating that the release kinetics could not only be influenced by the diffusion of the dissolved drug but that they could also be strongly impacted by the erosion of the hydrophilic excipients (PEG400). This is consistent with previous studies [38]. The release kinetics from the pellets from all formulations followed the first-order equation according to the *R* value for the smaller pellets (1 mm and 2 mm), demonstrating that the release of ibuprofen from SLA-printed pellets with an adequately small size is independent of hydrophilic excipients.

### 3.8. Stability of the Printed Pellets

**Figure 10:**
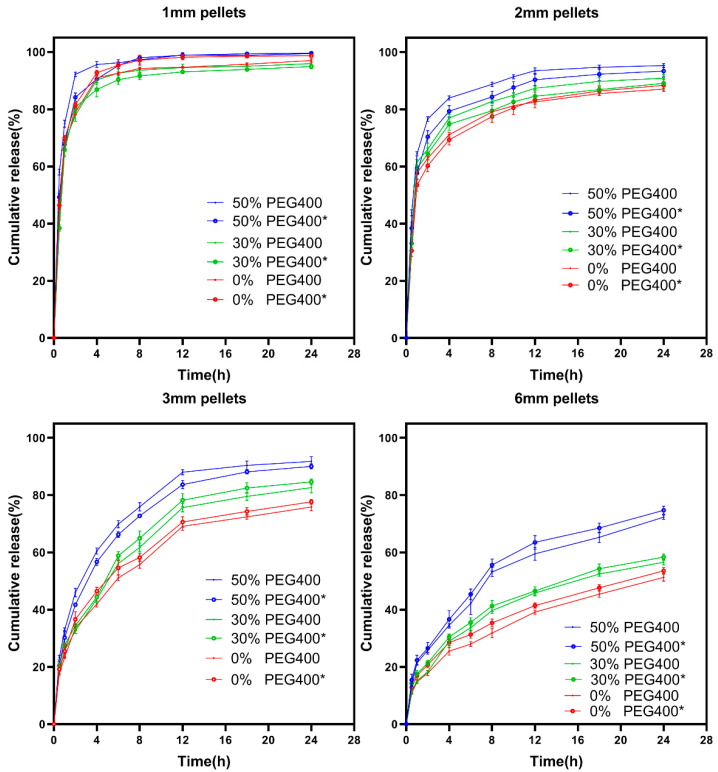
Drug release profile from different sized pellets with different PEG400 contents after stability tests (* = after storage at 40 °C, 75% RH for 1 month).

The release profiles of ibuprofen from SLA-printed pellets before and after storage were shown in Figure 10. The similarity factor *f*_2_ values are shown in Table 5. Those results indicate that the 3D-printed pellets with drug-loaded photocurable polymer resin exhibited excellent stability over 1 month at 40 °C/75% RH. It should be noted that the influence of the hydrophilic excipient (PEG400) on the stability of pellets with different sizes is not consistent, although this discrimination is very slight. For the smaller pellets (1 mm and 2 mm), increasing the ratio of PEG400 did cause an obvious reduction of the stability of the pellets, but there was a clear reduction of the similarity factor *f*_2_ values (Table 5), especially when increasing the PEG400 ratio to 50%. While such a correlation did not occur for the larger pellets (3 mm and 6 mm). The main reason for such results might be the difference of SA/V ratio (surface area divided by the volume) for the printed pellets with different sizes.

## 4. Conclusion

In this study, ibuprofen-loaded mini-sized pellets with a desirable hydrophilic excipient-independent drug release behavior and an adequate storage stability were successfully printed using the SLA 3D printing technique. Additionally, we found that the drug release rate from the SLA-printed oral dosages could be tuned by either adding the necessary hydrophilic excipients (such as PEG400) in the printing formulation, or by adequately reducing the size of the produced dosages, allowing for a sufficient specific surface area for drug release by diffusion. The printed pellets with an adequately small size (1 mm and 2 mm) showed a PEG400-independent release behavior, which implies that the drug release behavior from an SLA-printed pellet could be easily manipulated by modulating the size instead of by adjusting the printing formulation. Practically, the absence of the hydrophilic excipients in the printing formulation during the SLA process not only significantly improved the printability but also promoted the mechanical properties of the produced dosages, both of which could create tremendous benefits in terms of their industrialization and practical applications. However, hydrophilic excipients may still be necessary when there is a minimum size requirement for a synchronously printed dosage with fast drug release behavior. In this circumstance, the amount of the applied hydrophilic excipients must be strictly controlled for both acceptable printability and sufficient stability.

## Figures and Tables

**Table 1:** Composition of formulations prepared for printing.

Formulations	PEGDA600 (*w*/*w*, %)	PEG400 (*w*/*w*, %)	Ibuprofen (*w*/*w*, %)	TPO (*w*/*w*, %)
Formulation 1	89.5	0	10	0.5
Formulation 2	59.5	30	10	0.5
Formulation 3	39.5	50	10	0.5
Formulation 4	19.5	70	10	0.5

**Table 2:** Measured parameters of tartrazine content in printed pellets (*n* = 10).

Tartrazine Ratio	Model	0.00% ^1^	0.02%	0.04%	0.08%	0.10%	0.12%
Long D(mm)	2	4.00 ± 0.18	3.15 ± 0.10	2.95 ± 0.08	2.49 ± 0.05	2.21 ± 0.03	2.24 ± 0.06
Short D(mm)	2	2.10 ± 0.11	2.09 ± 0.09	2.09 ± 0.06	2.08 ± 0.10	2.03 ± 0.03	1.98 ± 0.04
Roundness	0	1.90	1.06	0.86	0.51	0.18	0.26
Picture	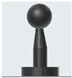	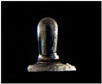	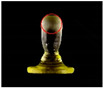	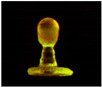	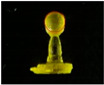	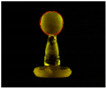	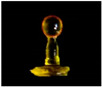

^1^ Based on the weight of PEGDA600.

**Table 3:** Drug content in photopolymer solution and printed mini pellets.

Formulations	Drug Content in Photopolymer Solution (%)	Drug Content in 1 mm Pellets (%)	Drug Content in 2 mm Pellets (%)
Formulation 1 (0% PEG)	98.0% ± 0.2	91.2% ± 1.6	89.1% ± 0.9
Formulation 2 (30% PEG)	97.5% ± 0.3	89.3% ± 1.2	87.6% ± 1.0
Formulation 3 (50% PEG)	97.1% ± 0.3	87.4% ± 0.9	86.3% ± 0.6

**Table 4:** Kinetic models of different drug release from different sized pellets.

Formulations	Zero Order	First Order	Higuchi	Peppas	Crowell	*n* Value
1 mm F1	0.292	0.990	0.587	0.937	0.656	-
1 mm F2	0.289	0.992	0.583	0.924	0.657	-
1 mm F3	0.207	0.995	0.485	0.947	0.526	-
2 mm F1	0.446	0.963	0.719	0.958	0.656	-
2 mm F2	0.451	0.952	0.721	0.958	0.703	-
2 mm F3	0.375	0.983	0.655	0.957	0.684	-
3 mm F1	0.746	0.951	0.952	0.982	0.871	0.36
3 mm F2	0.750	0.962	0.950	0.976	0.918	0.37
3 mm F3	0.654	0.966	0.900	0.967	0.928	0.31
6 mm F1	0.860	0.931	0.990	0.998	0.894	0.41
6 mm F2	0.807	0.946	0.974	0.992	0.853	0.38
6 mm F3	0.816	0.953	0.975	0.988	0.895	0.40

**Table 5:** Similarity factors of pellets with different size and formulation after stability tests.

Size	Formulation	*f*_2_ (Similarity Factors)
1 mm	F1	79.07
	F2	78.82
	F3	62.14
2 mm	F1	82.22
	F2	79.25
	F3	68.08
3 mm	F1	78.59
	F2	80.87
	F3	74.07
6 mm	F1	76.75
	F2	82.36
	F3	81.78

## Data Availability

Not applicable.
